# Grasp It Loudly! Supporting Actions with Semantically Congruent Spoken Action Words

**DOI:** 10.1371/journal.pone.0030663

**Published:** 2012-01-24

**Authors:** Raphaël Fargier, Mathilde Ménoret, Véronique Boulenger, Tatjana A. Nazir, Yves Paulignan

**Affiliations:** 1 L2C2-Institut des Sciences Cognitives, CNRS/UCBL, Université Claude Bernard Lyon1, Lyon, France; 2 Laboratoire Dynamique du Langage, UMR 5596 CNRS – Université de Lyon, Institut des Sciences de l'Homme, Lyon, France; University of Bologna, Italy

## Abstract

Evidence for cross-talk between motor and language brain structures has accumulated over the past several years. However, while a significant amount of research has focused on the interaction between language *perception* and action, little attention has been paid to the potential impact of language *production* on overt motor behaviour.

The aim of the present study was to test whether verbalizing during a grasp-to-displace action would affect motor behaviour and, if so, whether this effect would depend on the semantic content of the pronounced word (Experiment I). Furthermore, we sought to test the stability of such effects in a different group of participants and investigate at which stage of the motor act language intervenes (Experiment II). For this, participants were asked to reach, grasp and displace an object while overtly pronouncing verbal descriptions of the action (“grasp” and “put down”) or unrelated words (e.g. “butterfly” and “pigeon”).

Fine-grained analyses of several kinematic parameters such as velocity peaks revealed that when participants produced action-related words their movements became faster compared to conditions in which they did not verbalize or in which they produced words that were not related to the action. These effects likely result from the functional interaction between semantic retrieval of the words and the planning and programming of the action.

Therefore, links between (action) language and motor structures are significant to the point that language can refine overt motor behaviour.

## Introduction

Co-speech gesture is commonly thought to assist language production for the speaker and language comprehension for the listener [1], [2]. The interplay between action and language is also illustrated by studies revealing functional links between language processing and motor action. For instance, reading or listening to words that refer to bodily actions elicits activity in brain motor areas involved in action production ([3]–[11]; see [12] for a review). In line with these observations, Frak and collaborators showed that listening to manual-related action verbs increased grip force as early as 260 ms following word onset [13]. Behavioural evidence for reciprocal interactions between motor act and (action) language processing has also accumulated over the past few years [13]–[20]. Boulenger and colleagues [14] examined the impact of processing action words or concrete nouns on a concurrent reaching and grasping movement. Their results revealed that when an action word (but not a concrete noun) was perceived at movement onset, interference with the execution of the motor act was observed. Conversely, when the action word was perceived before movement onset, a facilitation effect appeared. The authors interpreted the interference effect as reflecting competition for shared resources between action and language functions (see [16] for related results). On the other hand, facilitation was seen as resulting from residual activity in motor/premotor structures due to the processing of the action word. Related to this, several studies also pointed out a functional link between words that refer to extrinsic and/or intrinsic object properties and the motor programs elaborated by participants to grasp the corresponding objects [21]–[23]. In their study, Gentilucci & Gangitano [23] asked volunteers to reach and grasp a rod on which the words “long” or “short” were printed. They found that the kinematics of reaching was affected by word presentation. Furthermore, during the initial movement phase, subjects automatically associated the meaning of the word with the distance to be covered and activated a motor program for a farther and/or a nearer object position (adaptation of the motor program). Other studies investigated language influence on motor processing with the so-called Action-Sentence Compatibility paradigm. This paradigm, which was developed by Glenberg and Kaschak [18], typically reveals a facilitation effect of the direction of motion implied by a sentence on the direction of a subsequent motor response (see also [24] for a review). Using this paradigm, Aravena and collaborators [25] recently revealed ERP-markers (evoked response potentials) of the cross-talk between processes involved in language comprehension and in motor acts. In this study, action-sentence compatibility pertained to hand-shape actions denoted by spoken sentences (e.g. “[…] Rocio applauded”) and hand shape motor responses (e.g. pressing a response button with the open hand). Coherent with previous findings [18], [26]–[28], Aravena et al. showed that participants were quicker to press the response button when the hand shape implied by the sentence was compatible with the hand shape required by the response. Moreover, larger amplitudes of motor potentials (MP) and reafferent potentials (RAP) were observed in the compatible condition, indicating a facilitation of the motor response when language and motor processes were congruent. Additionally, an N400-like effect emerged in the incompatible condition, suggesting that action-sentence incompatibility affected sentence comprehension at a semantic level. These studies are usually interpreted in the context of the embodied cognition framework which states that the representation of semantics involves, to some extent, sensory and motor brain networks. In turn, these representations are thought to modulate behaviour (see [24] for a review, but see also [29] for a range of embodied cognition theories).

However, so far, most studies have focused on the impact of language and/or action *perception* on motor and/or language processing, respectively. Here, we propose to test the impact of language *production* on overt motor behaviour. It has been shown previously that production of action words activates the motor cortex. In a Transcranial Magnetic Stimulation (TMS) experiment, for instance, Oliveri et al. [30] reported that activation of the left primary motor cortex increased during overt production of action words compared to non-action words, regardless of words' grammatical category. Since excitability of hand motor regions is selectively enhanced when action-related words are produced, overt production of action words could thus facilitate action execution.

The aim of the present study was to determine whether adding verbalization to the execution of a grasping-and-displacing movement significantly affects motor performance. More precisely, we sought to (1) shed light on a possible added value of verbalization on concurrent action execution and (2) determine whether this facilitation effect depends on the semantic content of the verbalization (action-related content vs. unrelated content). To this end, in a first experiment (Experiment I), participants were required to grasp and displace an object while verbalizing two words, one for each of the two sub-parts of the movement (i.e. the reach-to-grasp movement and the lift-to-displace movement). The pair of the to-be pronounced words were related to the performed movement (i.e. “grasp” and “put down”) or not (“butterfly” and “pigeon”). In this experiment, both movement and verbalization were self-paced. In a follow-up study (Experiment II), the same paradigm was applied with an additional condition in which the to-be pronounced words were related to actions performed with other effectors than the arm (e.g. “squat”). In Experiment II, word onsets and offsets were recorded simultaneously with the kinematic recordings.

Fine-grained kinematic parameters and movement duration served as dependent measures in both experiments. Since several studies reported a facilitation effect of (action) language on overt motor behaviour, we hypothesize that acceleration and velocity parameters will be sensitive to (action) word production. Increased amplitudes of acceleration as well as velocity peaks should be observed when verbalizing action-related words, but not other types of words.

## Materials and Methods

### 1. Experiment I

#### 1.1. Participants

Twenty-one healthy native French speakers (from 20 to 49 years old (mean age: 28.3), 13 females) took part in this experiment. All were right-handed (mean scores: 0.82 Edinburgh test [31]) and had normal or corrected-to-normal vision. In accordance with the principles of the Declaration of Helsinki, the study was approved by the Ethical Committee CPP Sud-Est II in Lyon. All participants gave their written informed consent.

#### 1.2. Procedure

**Figure 1:**
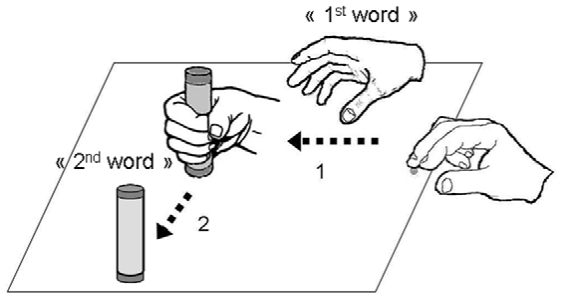
Experimental procedure. Participants (1) grasped the cylinder and (2) placed it to the left or to the right while verbalizing two instructed words (one for each movement).

Participants were asked to reach and grasp a small cylinder placed on a table in front of them and to lift and displace it to the left or to the right. Although the task was not a precision-task, two stickers indicated where the participants had to put the cylinder at the end of the second part of the movement. Participants had to reach the cylinder using a precision grip with all the fingers. While performing this action they verbalized pairs of words. The protocol is displayed in Figure 1. Participants were asked to pronounce the first word in relation to the reaching-to-grasp movement, and the second word in relation to the lifting-to-displace movement.

The experiment included 5 conditions:

- A **Non-Verbalization condition (NV)** (movement only)
- **A related-Action Verbal condition (rA)** (movement while verbalizing the following verbs (infinitive form) describing the performed action):
  “Attraper”/“Poser” (Grasp/Put down)
- Three **unrelated Verbal conditions (uV)** (movement while verbalizing animal names or number words unrelated to the performed action):
  «Papillon»/«Pigeon» (Butterfly/Pigeon)
  «Un»/«Deux» (One/Two)
  «Cent dix-neuf»/«Cent vingt» (119/120)

The three unrelated verbal conditions were included to determine if beyond verbalization per se, the semantic content (unrelated words or numbers vs. action-related words) affected action execution. The number of syllables of the to-be-produced words was controlled for each condition, except for the “One/Two” condition, which represents a common verbal cue during movement execution (e.g., in physical exercises or gymnastics). For each condition, there was only one pair of words to be pronounced. The 5 conditions were divided in 5 blocks of 20 trials each. Instructions were given at the beginning of each block and participants were requested to self-initiate their movements and verbalizations (i.e. there was no “Go” instruction). The order of the blocks was randomized and counterbalanced between participants.

At the beginning of each trial, participants were asked to place their right hand at a starting position on a table in front of them, with their right thumb and index finger held in a pinch grip position. Within each of the five blocks, participants alternated left and right placing movements. After each trial, the experimenter placed the cylinder back at the starting position. Participants were asked to perform all movements at a constant rhythm throughout the experiment.

### 2. Experiment II

#### 2.1. Participants

Sixteen healthy native French speakers (from 18 to 28 years old (mean age: 21.4), 9 females) took part in this experiment. All were right-handed (mean scores: 0.8 Edinburgh test [31]) and had normal or corrected-to-normal vision. In accordance with the principles of the Declaration of Helsinki, the study was approved by the Ethical Committee CPP Sud-Est II in Lyon. All participants gave their written informed consent. None of the volunteers for the second experiment had participated in the first experiment.

#### 2.2. Procedure

The procedure of this second experiment was similar to that of the first experiment. Participants were asked to reach and grasp a small cylinder placed on a table in front of them and to lift and displace it to the left or to the right. While performing this action they verbalized pairs of words (see Figure 1).

The experiment included 4 conditions:

- A **Non-Verbalization condition (NV)** (movement only)
- A **related-Action Verbal condition (rA)**
  “Attraper”/“Poser” (Grasp/Put down)
- An **unrelated-Action Verbal condition (uA)**
  «Accroupir»/«Courir» (Squat/Run)
- An **unrelated Verbal condition (uV)**
  «Aliment»/«Piment» (Food/Pepper)

An unrelated-Action Verbal condition was included to determine if the modulation of action execution was specific to the semantic content of the pronounced action words (action-related content vs. action-unrelated content). The number of syllables of the to-be-produced words was controlled for each condition. The 4 conditions were divided in 4 blocks of 20 trials each. Instructions were given at the beginning of each block. After a “ready” signal (i.e. a click), participants were requested to self-initiate their movements and verbalizations. The order of the blocks was randomized and counterbalanced between participants. The rest of the experiment was strictly identical to the first experiment.

### 3. Kinematic acquisition and analysis

An Optotrak 3020 (Northern Digital, Waterloo, Ontario) was used to record the spatial position of an active marker (infrared light-emitting diode), at a sampling rate of 250 Hz and with a spatial resolution of 0.1 mm. The marker, placed on the participant's wrist, characterized the reaching component [32], [33].

**Figure 2:**
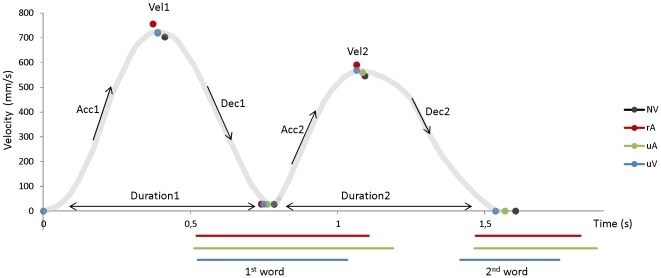
Kinematic profile of the movement. The gray curve represents a theoretical velocity curve of the movement. Acceleration peaks, Velocity peaks, Deceleration peaks and movement durations were measured. The reported dots correspond to the data obtained in Experiment II for each condition. Word durations are depicted as lines and onsets of word-production are locked to the movement (Red, green and blue lines represent rA, uA and uV conditions respectively).

Raw data was pre-processed using a second-order Butterworth dual pass filter (cut-off frequency, 10 Hz). Movements were then analyzed using Optodisp software (Optodisp - copyright INSERM-CNRS-UCBL, Thévenet et al., 2001). Kinematic parameters were assessed for each individual movement. We analyzed the amplitude of the wrist velocity peak (mm/s) as well as movement duration (milliseconds) for the two parts of the movement (i.e. grasping (Vel_1_ and Duration_1_) and lifting (Vel_2_ and Duration_2_)). For both movement parts, amplitude of the wrist acceleration (Acc_1_ and Acc_2_) and deceleration (Dec_1_ and Dec_2_) peaks were analyzed. Movement onset was determined as the first value of a sequence of at least eleven increasing points on the basis of the wrist velocity profile. For each part of the movement, wrist velocity peak was determined as the maximal value in the velocity profile (see Figure 2). Similarly, wrist acceleration and deceleration peaks were measured as the maximal and minimal values respectively in the acceleration profile. Kinematic parameters were determined for each individual trial and were then averaged for each participant and condition. Trials in which participants made errors were excluded from the analysis. For Experiment I only, a preliminary analysis was conducted to test significance between unrelated Verbal conditions (uV) using a repeated measures analysis of variance (ANOVAs) with 1 within-subject factor (3 levels). For both Experiments, analysis of variance (ANOVAs) with repeated measures including Condition as a within-subject factor (Experiment I: 3 levels; Experiment II: 4 levels) were used to assess significant differences between the conditions (Statistica 8, Statsoft Inc). Post-hoc tests (Newman–Keuls) were performed to distinguish the effects of each condition on the kinematic parameters. A significance level of p<0.05 was chosen. One participant was excluded from the analysis (Experiment I) due to significantly outlying kinematic values.

### 4. Word-production acquisition and analysis

In Experiment II, word onsets and offsets were recorded with a microphone headset. Regarding the analysis, each word onset was synchronized on each movement onset. Word durations were calculated for individual trials and were then averaged by condition and across subjects. Statistical analyses were similar to statistics for kinematics.

## Results

### 1. Experiment I

#### 1.1. 1^st^ part of the movement: “Reach-to-Grasp the object”

A preliminary analysis revealed no significant difference between the three unrelated Verbal conditions (uV) “Butterfly/Pigeon”, “One/Two”, “119/120” for any kinematic parameters. The ANOVA with the factor Condition did not reveal any significant differences for the velocity peak amplitude (Vel_1_: F(2,38) = 0.12; ns), the acceleration peak amplitude (Acc_1_: F(2,38) = 0.55; ns), the deceleration peak amplitude (Dec_1_: F(2,38) = 0.14; ns), or the movement duration (Duration_1_: F(2,38) = 0.19; ns) (see Table 1). The data for these conditions were thus collapsed and compared to the Non-Verbalization condition and the related-Action Verbal condition.

**Table 1:** Experiment I: Averaged values for all analysed parameters for each condition.

	1st part of the movement				2nd part of the movement			
Conditions	Vel1 (mm/s)	Duration1 (ms)	Acc1 (mm/s^2^)	Dec1 (mm/s^2^)	Vel2 (mm/s)	Duration2 (ms)	Acc2 (mm/s^2^)	Dec2 (mm/s^2^)
No verbalization (NV)	651±21	917±23	3319±192	−2588±145	385±17	902±25	2024±108	−1320±79
related Action Verbal (rA)	686±21	882±23	3639±232	−2877±171	404±22	902±25	2080±111	−1433±77
unrelated Verbal (uV)	661±20	915±24	3388±191	−2765±149	392±20	917±27	1989±106	−1341±66
*“Butterfly/Pigeon”*	662±20	910±23	3346±201	−2761±152	393±18	917±24	1965±96	−1342±57
*“One/Two”*	660±21	917±26	3379±181	−2787±151	394±19	907±28	2009±109	−1365±79
*“119/120”*	658±21	919±27	3440±213	−2749±161	389±20	928±33	1992±131	−1315±71

Mean values ± standard error of the mean (SEM).

**Figure 3:**
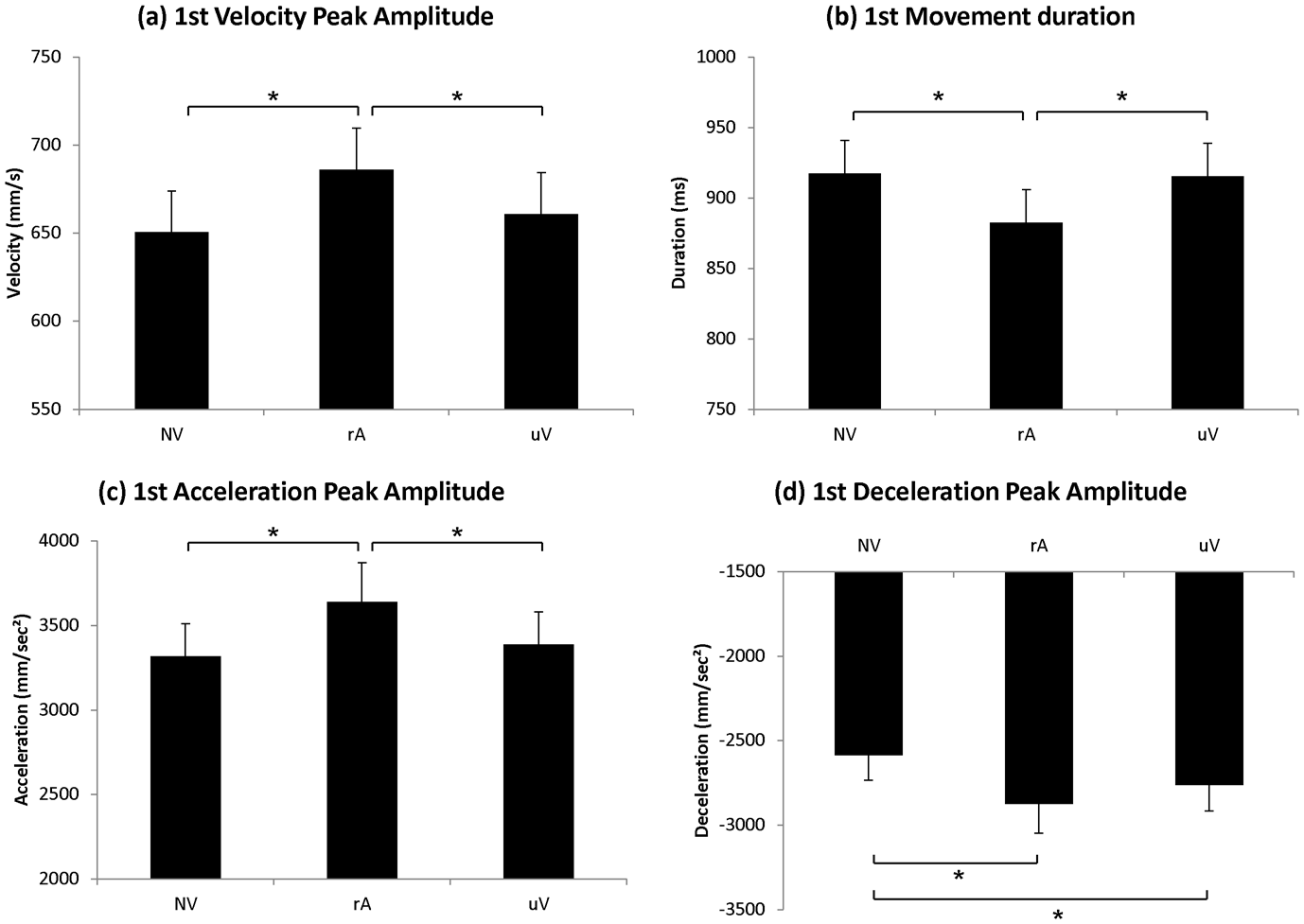
Experiment I: Kinematic parameters of the “Reach-to Grasp” movement. Mean values of (a) wrist velocity peak amplitude (b) movement duration (c) wrist acceleration peak amplitude and (d) wrist deceleration peak amplitude. Error bars represent standard error of the mean (SEM). *p<0.05.

The one-factor repeated-measures ANOVA (NV/rA/uV) showed a significant effect of Condition on the amplitude of the wrist velocity peak (F(2,38) = 6.21; p<0.005), on movement duration (F(2,38) = 3.927; p<0.03) and on the amplitude of the wrist acceleration peak (F(2,38) = 6.80; p<0.03). In the related-Action Verbal condition (rA), mean amplitudes of the wrist velocity peak and of the wrist acceleration peak were higher (Vel_1_: p<0.004; Acc_1_: p<0.003) compared to movements performed without verbalization (NV) and to movements in the unrelated Verbal condition (uV) (Vel_1_: p<0.02; Acc_1_: p<0.01). Mean movement duration was reduced in the related-Action Verbal condition (rA) (Duration_1_: p<0.04) compared to movements performed without verbalization (NV) and to movements in the unrelated Verbal condition (uV) (Duration_1_: p<0.03). All results are displayed in Table 1 and Figure 3. No other significant difference was observed.

Similarly, there was a significant effect of Condition on the amplitude of the wrist deceleration peak (F(2,38) = 9.61; p<0.0004). In the related-Action Verbal condition (rA) and in the unrelated Verbal condition (uV), mean amplitude of the wrist deceleration peak was stronger (Dec_1_: p<0.0004; Dec_1_: p<0.02 respectively) compared to movements performed without verbalization (NV). Results are displayed in Figure 3d. No other significant difference was observed.

Hence, when participants verbalized action words, the amplitudes of the wrist acceleration peak and of the velocity peak were increased by more than 5% while movement duration was reduced by nearly 4%. Amplitude of the deceleration peak was modulated in both related-Action and unrelated Verbal conditions. These results clearly indicate a facilitation effect of action word production on movement execution.

#### 1.2. 2^nd^ part of the movement: “Lift-to-Displace the object”

Similarly to the first part of the movement, the preliminary analysis revealed no significant difference between the three unrelated Verbal conditions (uV) “Butterfly/Pigeon”, “One/Two”, “119/120” for any kinematic parameters. The ANOVA with the factor Condition was not significant for the velocity peak amplitude (Vel_2_: F(2,38) = 0.35; ns), the acceleration peak amplitude (Acc_2_: F(2,38) = 0.24; ns), the deceleration peak amplitude (Dec_2_: F(2,38) = 0.92; ns) and movement duration (Duration_2_: F(2,38) = 0.98; ns) (see Table 1). The data for these conditions were thus collapsed and compared to the Non-Verbalization condition and the related-Action Verbal condition.

**Figure 4:**
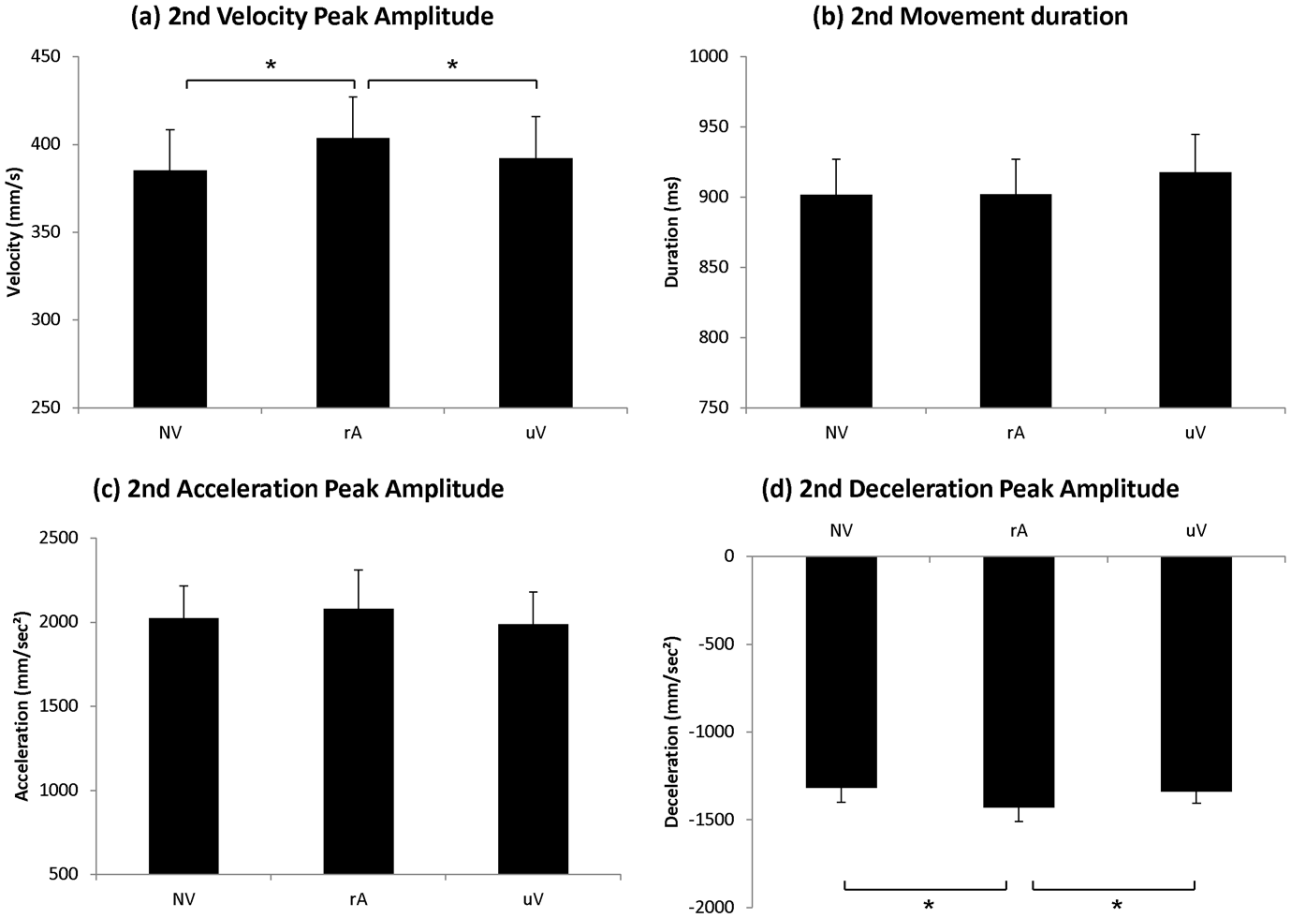
Experiment I: Kinematic parameters of the “Lift-to-Displace” movement. Mean values of (a) wrist velocity peak amplitude and (b) movement duration (c) wrist acceleration peak amplitude and (d) wrist deceleration peak amplitude. Error bars represent standard error of the mean (SEM). *p<0.05.

Figure 4 plots the corresponding results for the second part of the movement. The one-factor repeated-measures ANOVA (NV/rA/uV) showed a significant main effect of Condition on the wrist velocity peak amplitude (F(2,38) = 6.71; p<0.003). Post-hoc tests further revealed a significant difference between the related-Action Verbal condition (rA) and the two other conditions (NV: p<0.002 and uV: p<0.04), which did not significantly differ from each other.

Similarly, there was a significant effect of Condition on the amplitude of the wrist deceleration peak (F(2,38) = 6.62; p<0.003). In the related-Action Verbal condition (rA), mean amplitude of the wrist deceleration peak was accentuated compared to movements performed without verbalization (NV) (Dec_1_: p<0.004) as well as to movements in the unrelated Verbal condition (uV) (Dec_1_: p<0.01). No other significant difference was observed.

In contrast, there was no significant effect of the factor Condition on the duration of the lifting movement (Duration_2_) (F(2,38) = 6.71; ns) and on the wrist acceleration peak (F(2,38) = 1.69; ns).

When participants verbalized action words, the amplitudes of the velocity peak and of the deceleration peak were increased by more than 5%. In contrast with the first part of the movement, no effects on movement duration and acceleration peak were observed. Hence, even for the second part of the movement, these results suggest a facilitation effect of action word production on movement execution.

### 2. Experiment II

#### 2.1. 1^st^ part of the movement: “Reach-to-Grasp the object”

In this follow-up experiment, word-production onsets and offsets were recorded. The length of the first word was 584±22 ms for the related-Action Verbal condition (rA), 676±28 ms for the unrelated-Action Verbal condition (uA) and 507±17 ms for the unrelated Verbal condition (uV). The ANOVA revealed a significant effect of Condition on word-duration (F(2,30) = 61.2; p<0.0001). Despite strong differences in word durations, the onsets of word production locked to movement onset did not differ significantly between conditions (ANOVA: F(2,30) = 0.16; ns)). On average, the first word was produced 518±36 ms after movement onset for the rA condition, 512±43 ms after movement onset for the uA condition and 525±46 ms after movement onset for the uV condition (see Figure 2).

**Figure 5:**
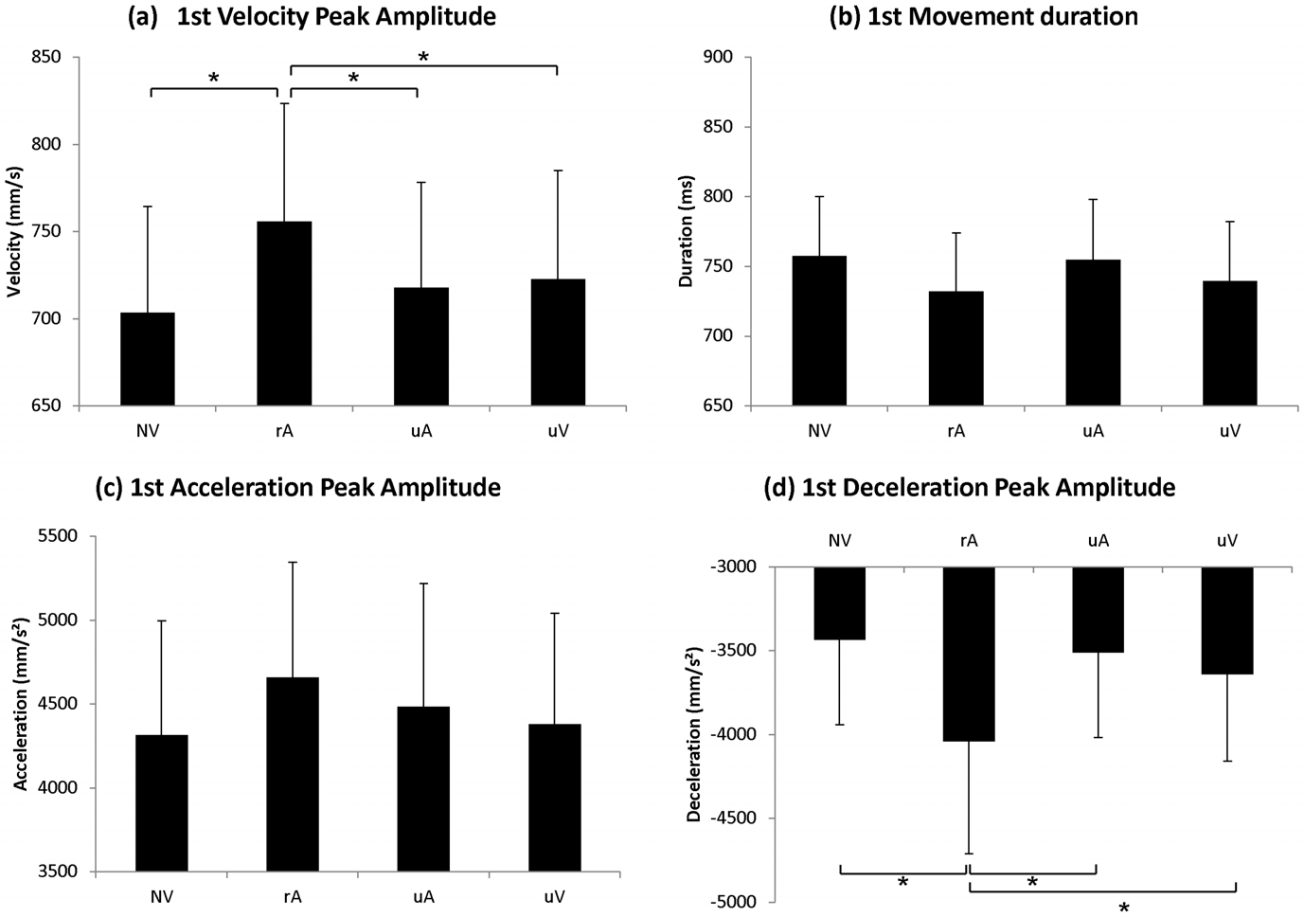
Experiment II: Kinematic parameters of “Reach-to Grasp” movement. Mean values of (a) wrist velocity peak amplitude (b) movement duration (c) wrist acceleration peak amplitude and (d) wrist deceleration peak amplitude. Error bars represent standard error or the mean (SEM). *p<0.05.

As to the kinematic measures, the one-factor repeated-measures ANOVA (NV/rA/uA/uV) showed a significant main effect of Condition on the amplitude of the wrist velocity peak (F(3,45) = 4.29; p<0.01). In the related-Action Verbal condition (rA), mean amplitude of the wrist velocity peak was higher than that of movements performed without verbalization (NV) (Vel_1_: p<0.006), movements performed in the unrelated-Action Verbal condition (uA) (Vel_1_: p<0.04), and those performed in the unrelated Verbal condition (uV) (Vel_1_: p<0.03). All results are displayed in Table 2 and Figure 5. No other significant difference was observed.

**Table 2:** Experiment II: Averaged values for all analysed parameters for each condition.

	1st part of the movement				2nd part of the movement			
Conditions	Vel1 (mm/s)	Duration1 (ms)	Acc1 (mm/s^2^)	Dec1 (mm/s^2^)	Vel2 (mm/s)	Duration2 (ms)	Acc2 (mm/s^2^)	Dec2 (mm/s^2^)
No verbalization (NV)	703±61	757±43	4316±681	−3437±503	546±25	824±44	2897±263	−2194±200
related Action Verbal (rA)	756±68	732±42	4657±689	−4044±667	592±25	805±48	3118±286	−2527±260
unrelated Action Verbal (uA)	718±60	755±44	4485±734	−3512±505	558±19	813±4	2887±209	−2348±187
unrelated Verbal (uV)	722±63	739±43	4379±663	−3643±515	566±21	797±44	2441±226	−2466±214

Mean values ± standard error of the mean (SEM).

For the amplitude of the wrist deceleration peak, we found a significant main effect of Condition (F(3,45) = 5.02; p<0.004). Mean amplitude of the deceleration peak was stronger in the related-Action Verbal condition (rA) than in the other conditions. Post-hoc analysis revealed that kinematic parameters in the rA condition were significantly different from those in the NV (Dec_1_: p = 0.005), the uA (Dec_1_: p<0.03) and the uV conditions (Dec_1_: p<0.01). No other significant difference was observed.

For the amplitude of the wrist acceleration peak, though the pattern of results was similar to Experiment I (see Figure 5c), the effect of Condition (NV/rA/uA/uV) did not reach significance (F(3,45) = 2.48; ns). Finally, there was no significant effect of Condition on movement duration (F(3,45) = 1.05; ns).

Similarly to what was observed in our first experiment, when participants verbalized action words, the amplitudes of the wrist velocity peak and deceleration peak were increased by more than 5%.

#### 2.2. 2^nd^ part of the movement: “Lift-to-Displace the object”

For the second part of the movement, the length of the word to be pronounced was 357±20 ms for the related-Action Verbal condition (rA), 414±24 ms for the unrelated-Action Verbal condition (uA) and 335±18 ms for the unrelated Verbal condition (uV). The ANOVA revealed a significant effect of Condition on word-duration (F(2,30) = 16.6; p<0.0001).

The same observation as for the first part of the movement was made for the second: Despite differences in word durations, the onsets of word production locked to the onset of the second part of the movement did not differ significantly between conditions (ANOVA: F(2,30) = 2.95; ns). On average, the second word was produced 726±42 ms after movement onset for the rA condition, 703±38 ms after movement onset for the uA condition and 668±43 ms after movement onset for the uV condition (see Figure 2).

**Figure 6:**
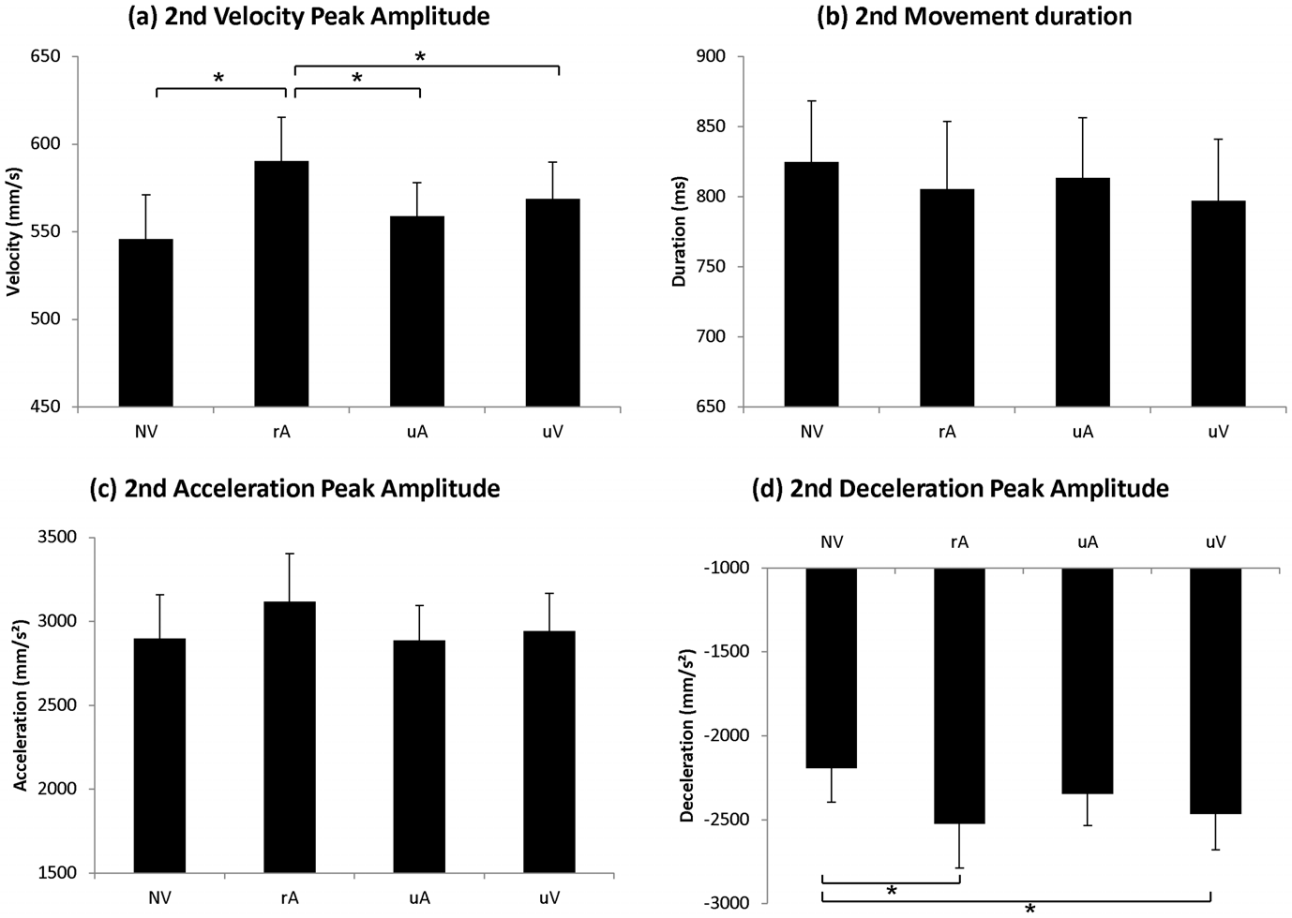
Experiment II: Kinematic parameters of the “Lift-to-Displace” movement. Mean values of (a) wrist velocity peak amplitude and (b) movement duration (c) wrist acceleration peak amplitude and (d) wrist deceleration peak amplitude. Error bars represent standard error of the mean (SEM). *p<0.05.

With regard to kinematic measures, mean amplitude of the velocity peak (Vel_2_) was stronger in the related-Action Verbal condition (rA) than in other conditions. Figure 6 plots the corresponding results. The one-factor repeated-measures ANOVA (NV/rA/uA/uV) showed a significant main effect of Condition (F(3,45) = 5.66; p<0.002). Post-hoc tests further revealed a significant difference between the rA condition and the three other conditions (NV: p<0.001; uA: p<0.02; uV: p<0.03), which did not significantly differ from each other (see Table 2).

We did not find any significant effect of Condition on the duration of the lifting movement (Duration_2_) (F(3,45) = 0.97; ns) or on the amplitude of the wrist acceleration peak (Acc_2_) (F(3,45) = 1.67; ns).

Concerning wrist deceleration peak amplitude, we found a significant main effect of Condition (F(3,45) = 5.56; p<0.003) on this parameter. Post-hoc analysis revealed that acceleration peaks in the related-Action Verbal condition (rA) were significantly different from those in the Non-Verbalization condition (Dec_1_: p<0.03). Post-hoc analysis also revealed a significant difference between unrelated Verbal condition and NV condition (Dec_1_: p<0.01). No other significant difference was observed.

Again, for the second part of the movement, the amplitude of the velocity peak was significantly higher when participants verbalized action-related words compared to the three other conditions. Note that for the amplitude of the deceleration peak, differences were observed between Non-Verbalization (NV) and related-Action Verbal (rA) conditions as well as between Non-Verbalization and unrelated Verbal (uV) conditions. Similarly to what was observed in the first experiment, we did not find any effect of word production on acceleration peak amplitude and movement duration.

## Discussion

The present study aimed at determining whether verbalizing during a grasp-to-displace movement would affect motor behaviour and, if so, whether this effect would depend on the semantic content of the pronounced word (Experiment I). Furthermore, we sought to test the stability of such effects in a different group of participants and to determine at which stage of the motor act language intervenes (Experiment II).

We show that amplitudes of the wrist acceleration peak (Experiment I), of the velocity and deceleration peaks (both experiments), and movement duration (Experiment I) were affected by verbalization of action-related words but not of other types of words. Despite slight changes that could be attributed to the modified procedure in Experiment II, the present effects are relatively stable considering that we observed the same results in two different groups of participants. In the following sections we discuss our results in more detail.

### 1. An in-depth interpretation of the kinematic effects

In both experiments, amplitudes of the wrist velocity peaks of the reach-to-grasp and lift-to-displace movements were increased when verbalizing action-related words compared to unrelated verbalization and the control condition (i.e. no verbalization).

In Experiment I, first wrist acceleration peak amplitude was enhanced in the related-Action Verbal condition only while first wrist deceleration peak amplitude was sensitive to any type of verbalization. The latter nonspecific effect was not observed in Experiment II, though deceleration peak of the second part of the movement showed less sensitivity than in Experiment I (enhancement for both rA and uV conditions but not for NV and uA conditions). Interestingly enough, in Experiment I, acceleration and deceleration were accentuated and movement duration reduced in the related-Action Verbal condition. However, we did not replicate this observation in Experiment II. A compensatory balance between acceleration and deceleration might explain the absence of effect on movement duration in Experiment II.

Rather than being two entirely independent movements, reach-to-grasp and lift-to-displace are subparts of a single unique action. In line with the idea of an action-chain mechanism involved in the selection of impending motor acts [34], both parts of the action are likely to be planned and programmed jointly. Cattaneo and colleagues [34] for instance, asked children either to reach and grasp a piece of food and then bring it to the mouth or to reach and grasp a piece of paper and place it in a container placed on their shoulder. By recording activity of the mouth-opening mylohyoid (MH) muscle, they revealed that participants showed an activation of their MH muscle already for the initial act of reaching (for food but not for paper). As concluded by the authors, muscles that mediate the action's final goal increase their activity as soon as the action starts [34] (see also [35] for similar observations for movements that differed in difficulty; or [36] for studies with monkeys). In line with this idea, it has been demonstrated that neurons in monkeys' F5 premotor area code for one particular type of movement (i.e. whole hand grasp vs. precision grip) [37], [38]. Such a motor repertoire would hold precise high-level description of the action [39], [40]. Hence, this assumption accounts for a semantic specificity of the organization of the motor system.

Altogether, these findings suggest that reach-to-grasp and lift-to-displace movements but also verbalization of the pair of words might all be prepared within the same period. Note that because of its attention-grabbing nature, it could have been expected that any verbalization during movement execution would enhance motor performance. Yet, our results show that unrelated action verbalization had no impact on the motor task. The effects reported in our experiments are thus likely to result from the functional interaction between semantic retrieval of the words and the planning and programming of the action.

### 2. General speech-associated effects on corticospinal excitability

Effects on specific kinematic parameters such as acceleration or velocity peaks reflect modulations of muscular contraction and thus corticospinal excitability. While in the present study the semantic impact of spoken words on motor behaviour was examined, previous studies more generally investigated speech-associated effects on motor system excitability [41]–[43]. Tokimura and colleagues [43], for instance, showed increased amplitude of electromyographic responses (EMG), recorded over right hand muscles, during reading aloud. Similarly, Meister and collaborators [41] asked participants to read aloud concrete words while hand and leg motor areas were stimulated. By applying TMS at various temporal intervals, the authors revealed an increase of motor evoked potentials (MEPs) amplitudes on right hand muscles for left motor cortex stimulation during reading. The authors did not report any effect on leg muscles (but see [44] for contrasting results). Altogether, these results show that linguistic production can impact corticospinal excitability. Note though that in our experiment, we did not find any effect of unrelated verbalization.

Coherent with these studies, Terao et al. [45] reported alternative hemispheric lateralization during cortical motor preparation of speech. By using TMS to temporarily suppress cortical functions, the authors aimed to look at the time course of activations in the sensorimotor cortices, supplementary motor area and cerebellum while participants prepared to produce a vocalization. They showed that cortical preparation for vocalization starts as early as 200 ms before voice onset, and also a mild left hemispheric predominance in the early phase [45].

Overall, hemispheric lateralization of speech-associated effects [42], [43], [45] and early timing of articulatory processes ([45]; see [46] for a review) are in agreement with the idea that our results cannot be attributed to exclusively motor effects due to the spatial proximity of the motor representation of the hand and the mouth [37], [38] or to articulatory schemes.

### 3. A well-documented cross-talk between motor action and language processes

The present findings are consistent with several previous studies showing that action words influence the execution of movements. Gentilucci and colleagues [47]–[49] investigated this issue from a “communicative” point of view. Instead of using object-directed actions, they asked participants to produce communicative movements and congruent related words, such as waving the hand to say “hello”. Bernardis & Gentilucci [48] and Barbieri et al. [47] found that gesture reinforced words, as reflected by enhancement of the voice spectra while, at the same time, they reported a reduction of arm peak velocity indicating an inhibition of gesture by word production. Relatedly, Chieffi et al. [49] instructed subjects to perform deictic movements (i.e. participants pointed “towards” them or “far” from them) while reading aloud a word congruent with the movement (e.g. a word that means “here” or “there” respectively). They also found reciprocal effects between gesture and language. Similarly to the previously reported experiments, an enhancement of voice spectra by gesture was observed. However, the effects of word production on movement parameters were opposite to those measured by Bernardis & Gentilucci [48]: when the pronounced word matched the movement, the latter was faster than when the word was incongruent. In order to explain the disparities between the previously mentioned results, we can hypothesize that effects of verbalization on movement depend on the goal of the task. In Bernardis & Gentilucci's study [48], velocity peak was reduced because the movement was performed to “assist” language (such as waving the hand to say “hello”). By contrast, in our study (and to a certain extent in the study by Chieffi et al. [49]), language assisted the motor action. In their recent study, Kritikos and colleagues [50] suggested that hand and finger positions should systematically change with the “spatial” meaning of a pronounced word. Participants had to reach the top or the bottom of a bar in response to the location of a word (synonyms of “up” and “down” such as “climbing” and “falling”). They found that word meaning modulated the trajectory of the movement. More than a simple effect of speech, the authors assumed that semantic coding (during articulation) influences the action.

### 4. At which stage does language intervene in the motor act?

It is well-known that various components of the motor program underlying a movement are computed prior to movement onset [51]–[53]. Interference between motor behaviour and (action) language processing may thus operate at any stage of the motor process. Boulenger et al. [14] and Nazir et al. [20] showed that perceived action words could transiently perturb the execution of an ongoing movement. The same team later provided evidence that the preparatory processes of a movement can also be influenced by language processing [15]. According to Dalla Volta et al. [16], modifications in velocity peaks (i.e. the main kinematic parameter resulting from the planning of the action) are indicative of an adjustment of the entire motor program. Coherent with this idea, several studies indicated that motor-related activity during language processing should result from anticipatory mechanisms [54], [55]. Facilitation/interference effects are differentially predicted according to the temporal relationship between action execution and language processing. Indeed, facilitation effects were observed when words were processed prior to movement onset, while interference effects occurred during simultaneous processing [14], [16], [56]. In a recent study, Chersi et al. [57], using a computational method, predicted such temporal-dependent facilitation and interference effects. In the present study, though the timing of semantic retrieval is difficult to assess (especially for self-paced spoken words), we assume that the passage from lexico-semantic processes to articulatory schemes is operated within 600 ms (see [45] for preparation of vocalization; [46] for a review on the neural correlates of language production). Given that the words were pronounced about 520 ms after the onset of the reach-to-grasp movement and 700 ms after the onset of the lift-to-displace movement, it is likely that retrieval of semantic representation of the action verbs tapped into the programming of the action.

### 5. Conclusion

Previous cognitive literature has paid surprisingly little attention to language production processes and their potential impact on motor behaviour. In our experiments, we show that spoken action-related words can support the motor act through the (positive) modulation of specific kinematic parameters. Although reach-to-grasp an object is largely an automatic and unconscious process [58], [59], its kinematics is sensitive to the semantic retrieval of action words. Since corticospinal excitability can be modulated by the preparation of action word production, verbalizing congruent action words should refine movement initiation and smoothness in patients who show selective motor deficits.
